# Waste-to-Resource Strategy to Fabricate Functionalized MOFs Composite Material Based on Durian Shell Biomass Carbon Fiber and Fe_3_O_4_ for Highly Efficient and Recyclable Dye Adsorption

**DOI:** 10.3390/ijms23115900

**Published:** 2022-05-24

**Authors:** Zhangzhen Cai, Qi Liu, Haoxin Li, Jingyi Wang, Guoyu Tai, Fan Wang, Jiangang Han, Yongli Zhu, Guangyu Wu

**Affiliations:** 1 Co-Innovation Center for the Sustainable Forestry in Southern China, College of Biology and the Environment, Nanjing Forestry University, Nanjing 210037, China; caizznjfu@126.com (Z.C.); liuqichem@126.com (Q.L.); lihaoxinnjfu@126.com (H.L.); wangjychem@126.com (J.W.); taigynj@126.com (G.T.); w1032365184@163.com (F.W.); lyly1262011@126.com (Y.Z.); 2 Guangxi Key Laboratory of Petrochemical Resource Processing and Process Intensification Technology, School of Chemistry and Chemical Engineering, Guangxi University, Nanning 530004, China

**Keywords:** biomass fibers, durian shell, magnetic composite, adsorption

## Abstract

Recently, metal–organic frameworks (MOFs), which are porous inorganic–organic hybrid materials consisting of metal ions (clusters or secondary building units) and organic ligands through coordination bonds, have attracted wide attention because of their high surface area, huge ordered porosity, uniform structural cavities, and excellent thermal/chemical stability. In this work, durian shell biomass carbon fiber and Fe_3_O_4_ functionalized metal–organic framework composite material (durian shell fiber-Fe_3_O_4_-MOF, DFM) was synthesized and employed for the adsorption removal of methylene blue (MB) from wastewater. The morphology, structure, and chemical elements of the DFM material were characterized by scanning electron microscope (SEM), X-ray diffraction (XRD), transmission electron microscope (TEM), and X-ray photoelectron spectroscope (XPS) techniques. Adsorption conditions such as pH, adsorption time, and temperature were optimized. The adsorption isotherm and kinetics results show that the adsorption process of DFM material to MB is more in line with the Freundlich model and pseudo-second-order kinetic model. Using these models, the maximum adsorption capacity of 53.31 mg/g was obtained by calculation. In addition, DFM material could be easily reused through an external magnet and the removal rate of MB was still 80% after five adsorption cycles. The obtained results show that DFM composite material, as an economical, environmentally friendly, recyclable new adsorbent, can simply and effectively remove MB from wastewater.

## 1. Introduction

With the development of industry, the increase of organic pollutants in wastewater will have various adverse effects on the environment, so more effective treatment methods need to be explored [1,2]. Methylene blue (MB) is an organic dye commonly used in the textile, leather, printing, and dyeing industries. Because of its high chroma and large organic content, if it is directly discharged into the surroundings without treatment, it will endanger the physical and mental health of the human body, causing people to have rapid heartbeat, nausea, and vomiting and, in serious cases, causing cancer. [3]. I methylene blue enters water, it will lead to poor light transmittance and affect the growth of plants and animals in water [4]. Common water-treatment technologies include flocculation precipitation, membrane separation, photocatalysis, biological methods, and ion exchange [5,6,7,8,9,10,11,12,13,14]. Compared with several other treatment technologies, the adsorption method has the advantages of high treatment efficiency, simple operation, no by-products, and recyclability, making it one of the important methods in pollutant treatment [15]. Commonly used adsorbents include activated carbon, molecular sieves, silicone, and zeolite [16], however, these adsorbents are difficult to recycle and the effect of reuse is poor [17,18]. So, designing a new adsorbent with a simple method, easy separation, and good chemical/physical stability is important.

Biomass is a general term for organisms that come directly or indirectly from various plants, with the most extensive source being agricultural and forestry waste [19,20,21].The common biomass treatment methods include incineration and burial. These treatment methods not only waste resources, but also cause other environmental pollution problems [22,23]. Durian shell is a type of agricultural and forestry waste. The main components are cellulose, hemicellulose, and lignin. It is a good material for the preparation of biomass adsorbents [24]. However, most of the common adsorbents prepared from durian shells have the disadvantages of large particle size and difficulty in recycling after adsorption [25,26,27]. Moreover, Fe_3_O_4_ shows excellent separate characteristics due to its magnetic properties [28]. Therefore, it has been widely used in wastewater treatment in recent years [29,30,31]. In previous studies, Sun et al. [32] prepared Fe_3_O_4_@C submicron rods by hydrothermal carbonization to remove MB in aqueous solution, but the larger particle size affects the comparison area and reduces the adsorption efficiency of MB. Rsjput et al. [33] synthesized Fe_3_O_4_ nanoparticles by co-precipitation to adsorb Pb^2+^. However, Fe_3_O_4_ nanoparticles are easy to agglomerate and the maximum adsorption capacity is only 53.11 mg/g. Therefore, it is necessary to study and prepare an adsorbent that can prevent Fe_3_O_4_ nanoparticles from agglomerating and has good pollutant removal effect.

Metal–organic framework materials (MOFs) based on their huge porosity, large specific surface area, adjustable pore size, etc. [34,35], are very broad prospects for various applications [36]. In recent years, various MOF materials have been applied to wastewater treatment. Abdi et al. [37] reported zeolite imidazolate framework (ZIF-8) nanocomposites prepared with graphene oxide (GO) and carbon nanotubes (CNT) as substrates to remove malachite green (MG) from colored wastewater. Lin et al. [38] synthesized a zirconium-based MOF (UiO-66-NH_2_) to remove fluoride in wastewater and the crystal structure of UiO-66-NH_2_ remained intact during the adsorption process. Efome et al. [39] synthesized water-based metal organic framework particles (Zr-808) on the surface of polyacrylonitrile (PAN) nanofibers, and the maximum adsorption capacities for heavy metal ions (Cd^2+^ and Zn^2+^) in aqueous solution were 225.05 and 287.06 mg/g, respectively. Although the MOF material can effectively remove pollutants in water, it is difficult to separate and recover quickly at the end of adsorption, which limits its practical application in wastewater treatment. In our work, fibers were extracted from durian shell and Fe_3_O_4_ nanoparticles were loaded onto its surface, which not only realized the resource reuse of durian shell, but also effectively reduced the agglomeration of Fe_3_O_4_ nanoparticles. At the same time, the combination of Fe_3_O_4_ nanoparticles and MOF materials can effectively solve the problem that MOF materials are difficult to separate from aqueous solution. In this paper, the durian shell fiber–Fe_3_O_4_ MOF composite was synthesized by a step-by-step method to remove methylene blue (MB) from wastewater. The structures were characterized by SEM, TEM, XRD, and XPS techniques. The effects of adsorption time, initial concentration, temperature, and pH value on the adsorption process, and the adsorption kinetics, isotherm, and thermodynamics of MB were studied.

## 2. Results and Discussion

### 2.1. SEM and TEM Analysis

**Figure 1:**
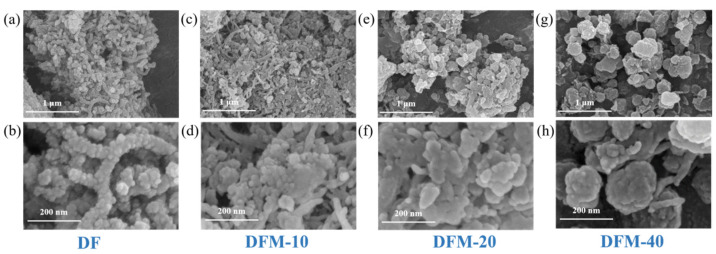
SEM images of DF (**a**,**b**), DFM-10 (**c**,**d**), DFM-20 (**e**,**f**), and DFM-40, and (**g**,**h**) samples at different magnifications.

**Figure 2:**
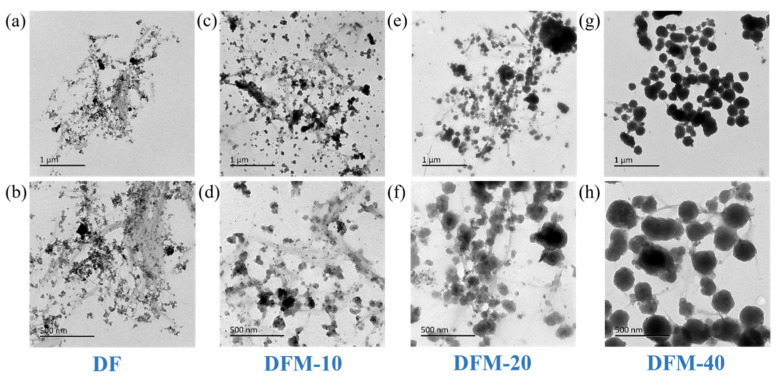
TEM images of DF (**a**,**b**), DFM-10 (**c**,**d**), DFM-20 (**e**,**f**), and DFM-40, and (**g**,**h**) samples at different magnifications.

The morphology and structure of DF, DFM-10, DFM-20, and DFM-40 samples at different magnifications were observed by SEM and TEM and the result are shown in Figure 1, Figure 2 and Figure S1. The width of durian shell fiber was around 10 µm and the surface of durian shell fiber was relatively smooth (Figure S1). As can be seen from DF images, the particle size of Fe_3_O_4_ was uniform and the magnetic Fe_3_O_4_ nanoparticles were successfully loaded onto the surface of durian shell fibers. From SEM results of DFM-10, DFM-20, and DFM-40, the magnetic Fe_3_O_4_ nanoparticles were successfully wrapped with a layer of MOF shell. After 10 cycles, sample DFM-10 showed a rough surface. The thickness of the MOF shell gradually increased with the increase in cycle numbers. However, after 20 and 40 cycles, the thickness of the MOF shell had a very significant increase compared with that after 10 cycles and the magnetic Fe_3_O_4_ nanoparticles appeared cross-linked and agglomerated. From TEM images, we found that the Fe_3_O_4_ nanoparticles were surrounded by MOF shells. As the number of cycles increased, the thickness of the MOF shell gradually increased.

**Figure 3:**
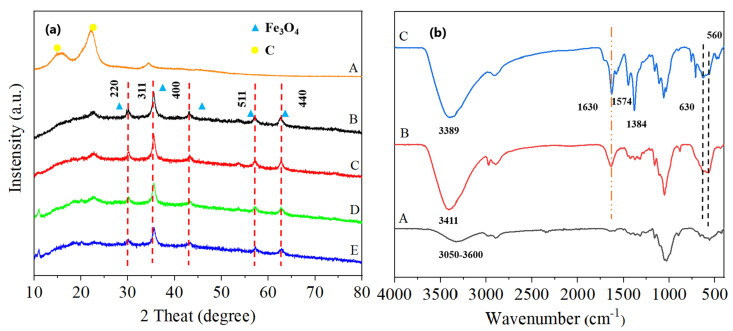
(**a**) XRD patterns of durian shell fiber (A), DF (B), DFM-10 (C), DFM-20 (D), and DFM-40 (E) and (**b**) FTIR spectra of durian shell fiber (A), DF (B), and DFM-10 (C).

The XRD peaks of the durian shell fiber and the prepared DF, DFM-10, DFM-20, and DFM-40 are shown in Figure 3a. As can be seen from (A), the crystal lattice of the durian shell fibers had peaks of 15.9°and 21.88°, which is consistent with previous literature reports [40]. The X-ray diffraction pattern of DF in (B) shows the result of diffraction peaks at 2θ values of 30.1°(220), 35.5°(311), 43.2°(400), 57.1°(511), and 62.8°(440), which is consistent with the standard XRD (JCPDS card, file No. 89-4319) data of Fe_3_O_4_ with a face-centered cubic structure reported in [41]. DFM-10, DFM-20, and DFM-40 also had the same diffraction peaks (C–E), indicating that the lattice structure of Fe_3_O_4_ will not be destroyed after many cycles. At the same time, with the loading of Fe_3_O_4_ and the MOF structure of the synthetic durian shell fiber, the diffraction peaks at 13° to 25° were significantly weakened. FTIR spectra of durian shell fiber (A), DF (B), and DFM-10 (C) materials are shown in Figure 3b.The peaks at 560 and 630 cm^−1^ could be ascribed to the stretching vibration of Fe-O in Fe_3_O_4_, indicating the existence of Fe_3_O_4_ in the materials. Additionally, peaks at 1634 and 1421 cm^−1^ were observed in the products of DFM-10, which, respectively, corresponded to C=C and C-O vibrations, confirming the presence of carboxylate groups. The carboxylate group in H_3_BTC combines with Fe^3+^ ions to coordinate to form MOF. In this way, the growth of MOF shell proceeded on the DF. The vibration bands corresponded to the -O-C-O- groups around 1574 cm^−1^ and 1384 cm^−1^, indicating the presence of the dicarboxylate within the compound. The strong stretching-vibration bands in the range of 3050–3500 cm^−1^ belonged to -OH and -NH_2_.

**Figure 4:**
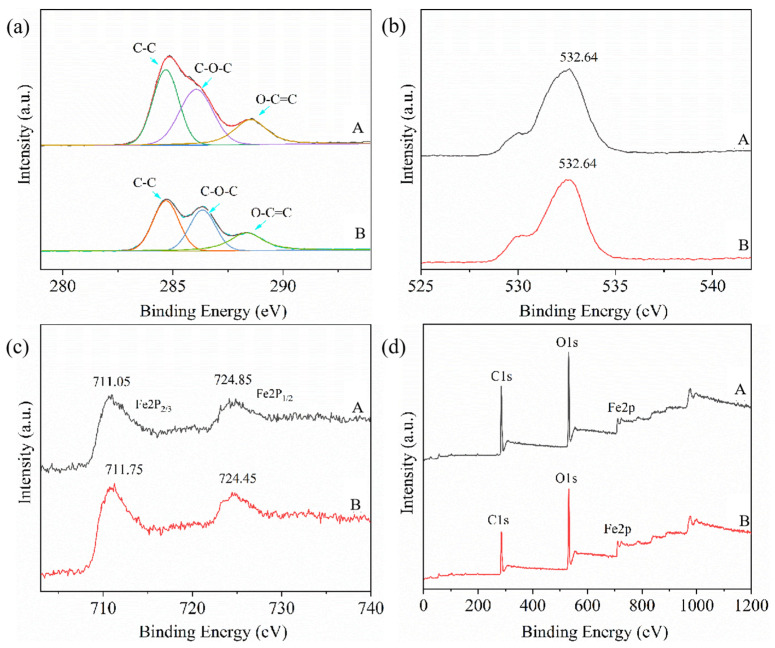
XPS spectra of C 1s (**a**), O 1s (**b**), Fe 2p (**c**), and broad scans (**d**) of DFM-10 before (A) and after (B) the adsorption of MB.

XPS was used to observe the changes in the chemical valence state of the elements before and after the adsorption of MB by DFM-10, and the spectra of C 1s, O 1s, Fe 2p, and broad scans are showed in Figure 4. Figure 4a shows the C 1s spectrum, which had three main peaks, namely C-C (284.8 eV), C-O-C (268.1 eV), and O-C= O (287.7 eV). Figure 4b shows the O 1s spectrum. The peak value was at 532.64 eV binding energy and the peak value did not change before and after adsorption, indicating that no oxidation–reduction reaction was involved in the adsorption process. Figure 4c shows the spectrum of Fe2p. It can be seen that the peaks at 711.05 eV and 724.85 eV represent Fe2p3/2 and Fe2p1/2, respectively, which indicates that Fe^2+^ and Fe^3+^ co-exist in the synthesized DFM-10. The total distribution energy spectrum of each element is shown in Figure 4d, from which it can be seen that there was little change before and after adsorption, indicating that the synthesized DFM-10 is a stable material. there was no obvious change in the characteristic peaks of the material before and after adsorption (A and B), indicating that DFM-10 was a stable material.

### 2.2. Adsorption Experiments

#### 2.2.1. Effect of Different Concentrations

**Figure 5:**
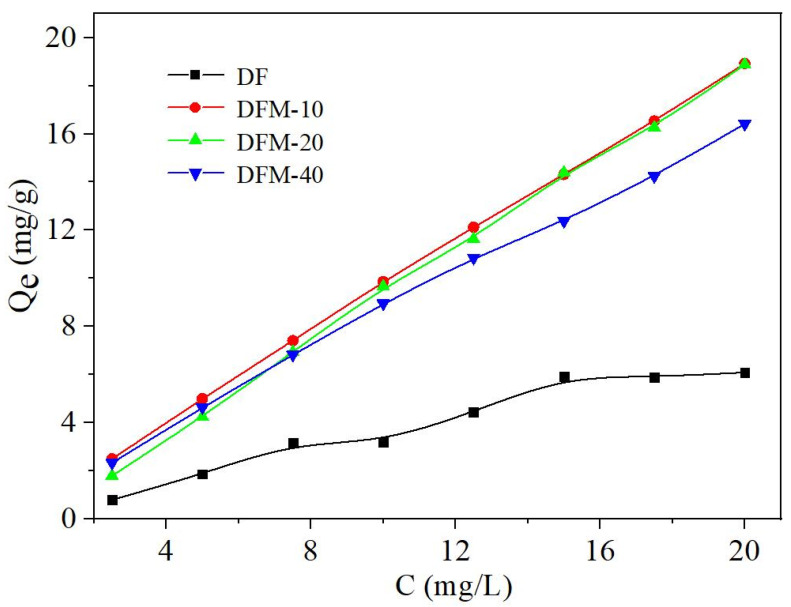
Adsorption effects of DF, DFM-10, DFM-20, and DFM-40 on MB at different concentrations.

To select a more suitable adsorbent, the adsorption performance of DF, DFM-10, DFM-20, and DFM-40 were evaluated. The adsorbent (5 mg) was mixed with MB solutions of different initial concentrations (5 mg/L, 10 mg/L, and 15 mg/L), and placed in constant-temperature oscillator at 25 °C. After a reaction time of 3 h, the adsorbent material was collected with a magnet. As shown in Figure 5, compared with DFM-0, the adsorption capacities of DFM-10, DFM-20, and DFM-40 were significantly improved, indicating that the introduction of a MOF structure significantly improve the adsorption performance of the material. The adsorption capacities of DFM-10 and DFM-20 to different concentrations of MB were basically the same, but the adsorption capacity of DFM-40 to MB was slightly lower than those of DFM-10 and DFM-20. Combined with SEM and TEM characterization, the reason may be that as the number of cycles increased, the thickness of the MOF shell gradually increased, and the MOF shell outside the adjacent magnetic Fe_3_O_4_ nanoparticles underwent crosslinking and agglomeration, which prevented the MB molecules from diffusing into the material, resulting in a decrease in adsorption effect. Therefore, DFM-10 was selected as the adsorbent, and its adsorption isotherm model, adsorption kinetics, and adsorption thermodynamics for MB were studied.

#### 2.2.2. Effect of pH and Ionic Strength

**Figure 6:**
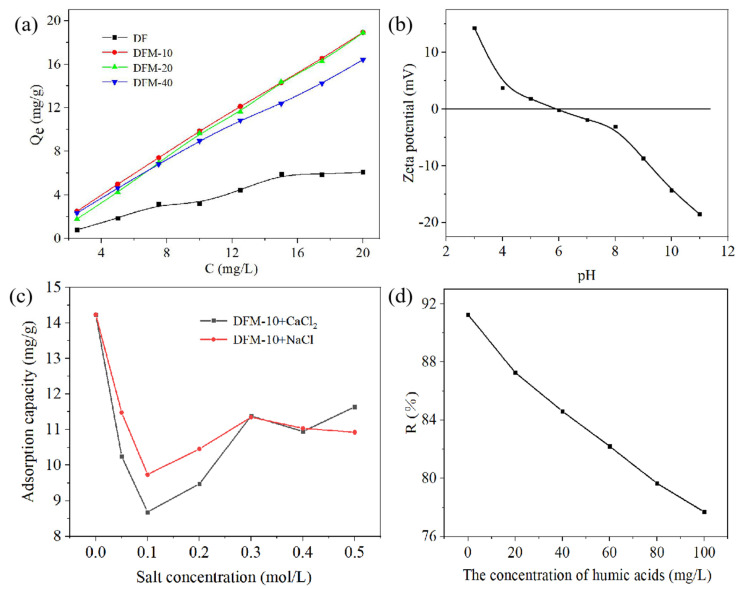
(**a**) Adsorption effects of DF, DFM-10, DFM-20, and DFM-40 on MB at different pH values, (**b**) zeta potential of DFM-10 adsorbent, (**c**) effect of ionic strength on the adsorption capacity, and (**d**) effects of humic acids solution concentration on MB removal percent.

pH plays a significant role in the process of adsorption. Therefore, the adsorption capacity of DF, DFM-10, DFM-20, and DFM-40 under different pH was studied, and the results can be seen in Figure 6a. It can be seen from the figure that as the pH value rose from 2 to 12, the adsorption capacity of DFM-0 on MB gradually increased. This was because of the presence of a large amount of H^+^ in the particles at low pH solutions, which led to the protonation of the Fe_3_O_4_ nanoparticle surface. however, the MB molecule was positively charged, so electrostatic repulsion occurred between the two, resulting in a small amount of adsorption With the gradual increase of pH, the surface of nano-Fe_3_O_4_ gradually deprotonated, so the adsorption capacity of MB gradually increased. However, the adsorption amount of MB on DFM-10, DFM-20, and DFM-40 did not change significantly with the increase of pH. This shows that the electrostatic interaction between DFM-10 (or DFM-20, DFM-40) and MB during the adsorption process was not the main factor affecting the adsorption effect. The larger adsorption capacity of DFM-10, DFM-20, or DFM-40 on MB may be attributed to the porous structure of the adsorbent itself, the van der Waals force and hydrogen bonding between the -OH and -COOH groups on the surface of DFM-10 (or DFM-20, DFM-40) and the MB molecule. Studying the influence of pH on the adsorption capacity provides an important reference for the practical application of materials. Therefore, considering comprehensive considerations, pH = 9 was selected in the next experiment. As can be seen from Figure 6b, the isoelelctric point(pHpzc) of DFM-10 was at 5.9, suggesting that the surfaces of the prepared DFM-10 adsorbents were positively charged at pH < 5.9 and the negatively charged at pH > 5.9.

In this work, NaCl and CaCl_2_, two common salts, were selected to judge the effects of ionic strength on the process of MB adsorption on DFM-10. As shown in Figure 6c, with the increase of salt concentration from 0 to 0.1 mol/L, the adsorption capacity decreased initially. This is because ionic strength reduced the surface charge of DFM-10 and MB, resulting in the decrease of adsorption capacity. When the concentration increased from 0.1 to 0.3 mol/L, the adsorption capacity increased because the salt may have reduced the solubility of MB in water. The decrease of solubility promoted the diffusion of more MB molecules to the adsorbent surface, thus improving the adsorption capacity. When the concentration of NaCl and CaCl_2_ was at 0.3mol/L, these two salts showed the same effect on adsorption capacity. From 0.3 mol/L to 0.5mol/L, the adsorption capacity showed a slight fluctuation. Although the adsorption capacity was reduced, the reduction was only 23.12%. Therefore, the results showed that the prepared DFM-10 adsorbent had potential application prospects in the treatment of wastewater containing MB.

The competitor experiments were performed towards humic acids under the optimal adsorption condition (adsorbent dosage: 5 mg; initial concentration of MB: 20 mg/L (10 mL); pH: 6; temperature: 25 °C; adsorption time: 3 h), and the results were shown in Figure 6d. As can be seen from Figure 6d, with increasing concentration of humic acids, the MB removal percent decreased. This could have been the result of the competitive effect between humic acids and MB molecule by electrostatic adsorption. However, the MB removal percent decreased by only 18.09% although the concentration of 100 mg/L for humic acids was far higher than that in a realistic water environment, indicating that the adsorbents have the potential to be employed for MB adsorption.

#### 2.2.3. Effect of Time and Initial Concentration of MB

**Figure 7:**
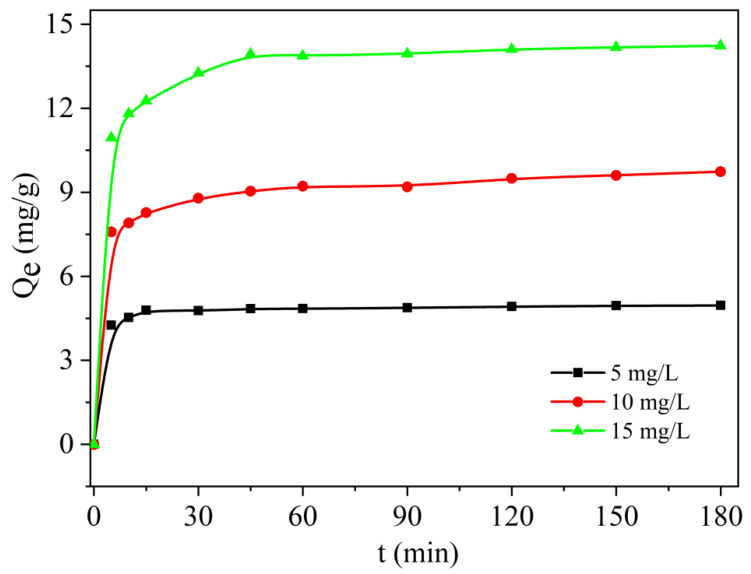
The relationship between adsorption amount and time of MB adsorbed by DFM-10 at different concentrations.

We added 5 mg of adsorbent to 15 mL of MB solution at concentrations of 5 mg/L, 10 mg/L, and 15 mg/L, and, after ultrasonic dispersion, placed the solutions in a constant-temperature shaker at 25 °C and vibrated for adsorption. The effects of different initial concentrations and adsorption time on the adsorption performance of DFM-10 were studied with time-interval sampling, and the results are shown in Figure 7.

It can be seen that with the increase of initial concentration, the adsorption amount of MB by DFM-10 also gradually increased. The adsorption process of MB on DFM-10 can be divided into two stages. The first stage was within 0–15 min after the beginning of adsorption. When the adsorbent is added, MB molecules quickly combined with the adsorption sites on the surface of DFM-10, and the adsorption capacity increased rapidly. With the passage of time, the adsorption sites were gradually occupied, which slowed down the diffusion rate of MB molecules into the adsorbent. Therefore, the increase of adsorption amount gradually slowed until the adsorption sites were completely occupied, an adsorption saturation state was reached, and the adsorption amount did not increase.

### 2.3. Adsorption Isotherms

To study the adsorption performance of DFM-10 to MB and their interaction pathways, Langmuir, Freundich, Temkin, and Dubinin–Radushkevich adsorption isotherm models were used. [42]. The Langmuir isotherm model assumes that only a single layer of adsorption occurs on the limited active sites on the surface of the adsorbent, and there is no interaction between adjacent sites [43]. The Freundich isotherm model assumes that the adsorption process is a multilayer adsorption, and there are interactions between adsorbate molecules [44]. The Temkin isotherm model considers the interaction between adsorbate and adsorbent. The Temkin model assumes that the adsorption heat of all molecules in the layer will decrease linearly with the accumulation of adsorbed molecules on the adsorbent surface. The Dubinin–Radushkevich isotherm model is an empirical model initially conceived for the adsorption onto micropore solids following a pore-filling mechanism. It is generally applied to express the adsorption mechanism with a Gaussian energy distribution onto a heterogeneous surface [21].

The Langmuir, Freundic, Temkin, and Dubinin–Radushkevich isotherm models are represented by the following equations:

(1) $$\mathrm{The}\;\mathrm{Langmuir}\;\mathrm{isotherm}\;\mathrm{model}\;\mathrm{equation}:\;\frac{C_{e}}{Q_{e}}=\frac{1}{Q_{L}K_{L}}+\frac{C_{e}}{Q_{L}}$$

(2) $$\mathrm{The}\;\mathrm{Freundich}\;\mathrm{isotherm}\;\mathrm{model}\;\mathrm{equation}:\;\ln Q_{e}=\ln K_{F}+\frac{1}{n}\ln C_{e}$$

(3) $$\mathrm{The}\;\mathrm{Temkin}\;\mathrm{isotherm}\;\mathrm{model}\;\mathrm{equation}:\;Q_{e}=\left(\frac{RT}{b_{T}}\right)\ln K_{T}+\left(\frac{RT}{b_{T}}\right)\ln C_{e}$$

(4) $$\mathrm{The}\;\mathrm{Dubinin}–\mathrm{Radushkevich}\;\mathrm{isotherm}\;\mathrm{model}\;\mathrm{equation}:\;\ln Q_{e}=\ln Q_{m}-K_{DR}\varepsilon^{2}$$

(5) $$\varepsilon =RT\ln\left[1+\frac{1}{C_{e}}\right]$$

where *Q_e_* (mg/g) shows the adsorption capacity at equilibrium and *Q_L_* (mg/g) is the theoretical maximum adsorption capacity. *C_e_* (mg/L) represents the concentration when the adsorption equilibrium is reached and *K_L_* is the Langmuir constant (L/mg). *K_F_* is the Freundlich constant (mg^1−1/n^ · L^1/n^/g). *1/n* is the parameter characterizing the energy heterogeneity of the adsorption surface. *b_T_* is the Temkin constant related to heat of adsorption (J·g/mol·mg). *K_T_* is the Temkin isotherm equilibrium binding-energy constant (L/mg). *R* is the gas constant (8.314 J/mol·K). *T* is the absolute temperature (K). *K_DR_* is the constant related to the adsorption energy (mol^2^·J^2^). *ε* is the adsorption potential (J/mol) [45,46].

**Figure 8:**
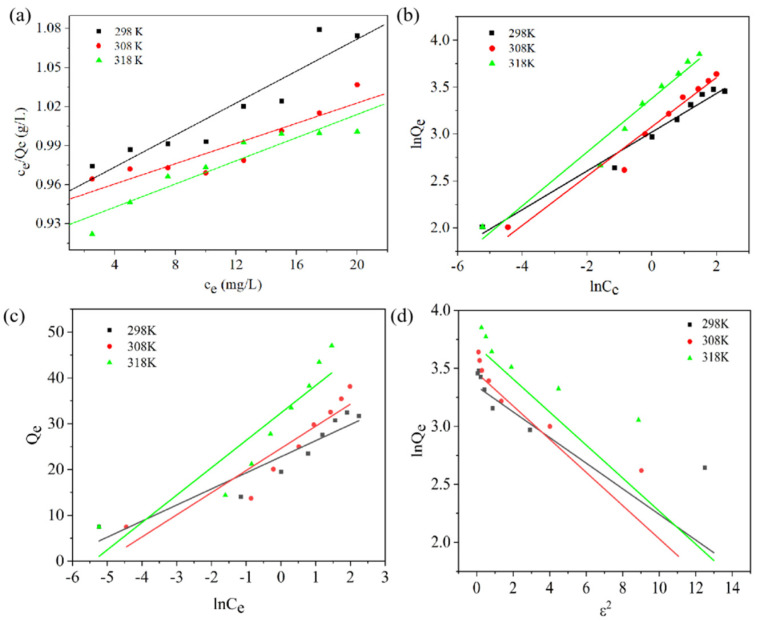
Langmuir (**a**), Freundich (**b**), Temkin (**c**), and Dubinin–Radushkevich (**d**) isotherms of MB adsorption on DFM-10.

The Langmuir, Freundich, Temkin, and Dubinin–Radushkevich isotherm models of MB adsorption by DFM-10 at different temperatures (298 K, 308 K, and 318 K) are shown in Figure 8, and the corresponding fitting parameters are listed in Table 1. It can be seen that the *R^2^* of the Langmuir isotherm fitting model was in the range 0.9791–0.9948, and the fitting curves was basically consistent with the experimental plots. The *R*^2^ of Freundich, Temkin, and Dubinin–Radushkevich isotherm models were in the ranges 0.9570–0.9766, 0.8447–0.8910, and 0.8567–0.8811, respectively, and the fitting curves were inconsistent with the experimental data. The correlation coefficients of the isotherms were in the order Langmuir > Freundich > Temkin > Dubinin–Radushkevich. The above results indicate that the Langmuir isotherm model was more appropriate for fitting the experimental data than other three models. In addition, with the increase of temperature, the adsorption amount also gradually increased, indicating that heating was conducive to the adsorption process. Monomolecular layer adsorption could have played a vital role, and the maximum adsorption capacities were 53.31 mg/g at temperature of 318 K. In the case of the Freundich isotherm model, the constant of Freundlich adsorption (*K_F_*) increased with increasing adsorption temperatures. High values of *K_F_* indicated that there was very good interaction between DFM-10 and MB.

### 2.4. Adsorption Kinetics

To calculate the adsorption kinetics of DFM-10 adsorbing MB, the experimental data were analyzed through pseudo-first-order, pseudo-second-order, and intraparticle diffusion kinetic models. The pseudo-first-order, pseudo-second-order, and intraparticle diffusion kinetic models are expressed by the following equations [47]:

(6) $$\ln\left(Q_{e}-Q_{t}\right)=\ln Q_{e}-K_{1}t$$

(7) $$\frac{t}{Q_{t}}=\frac{1}{K_{2}Q_{e}^{2}}+\frac{t}{Q_{e}}$$

(8) $$Q_{t}=K_{p}t^{\frac{1}{2}}+C$$

where *Q_e_* is adsorbed by adsorbents at equilibrium and *Q_t_* (mg/g) is the amount of MB adsorbed at time *t*. *K*_1_ (min^−1^), *K*_2_ (g mg^−1^ min^−1^), and *K_p_* (mg g^−1^ min^1/2^) are the rate constants of adsorption for the pseudo-first-order, pseudo-second-order, and intraparticle diffusion kinetic models.

**Figure 9:**
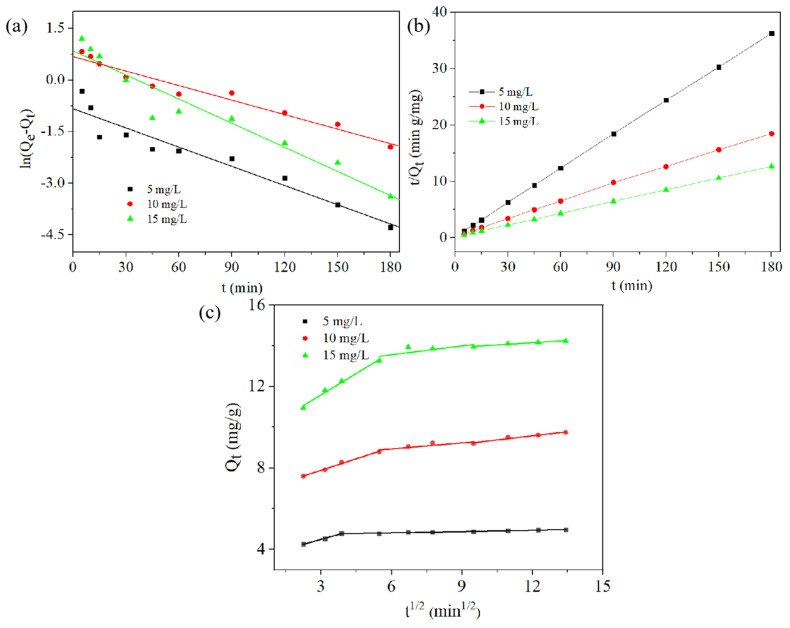
Pseudo-first-order (**a**), pseudo-second-order (**b**), and intraparticle diffusion (**c**) kinetic models fitting curves of MB.

Under the same temperature and dosage, different concentrations (5, 10, and 15 mg/L) of MB solution were selected to study the adsorption effect of DFM-10 on MB at different times. The fitting curves of pseudo-first-order (a), pseudo-second-order (b), and intraparticle diffusion (c) kinetic models of MB were obtained by data analysis and fitting as shown in Figure 9, and the corresponding fitting parameters are listed in Table 2. It can be seen from the fitting results that at different concentrations, the *R^2^* (0.9998, 0.9998, 0.9998) of the pseudo-second-order kinetic fitting model of DMF-10 for MB adsorption was greater than that of the pseudo-first-order kinetic fitting model (0.6817, 0.8401, 0.9523) and intraparticle diffusion kinetic model. This indicates that the adsorption process of DMF-10 to MB is more complex in the pseudo-second-order kinetic model, and the adsorption process is dominated by chemical adsorption. With the increase of initial concentration, the rate constant *K_2_* of the pseudo-second-order kinetic model decreased gradually. This shows that with the increase of initial dye concentration, the adsorption rate gradually slowed down, and the adsorption rate was faster at low concentration. The intraparticle diffusion kinetic curves were treated to test whether internal particle diffusion or external diffusion was the rate-limiting step. The intraparticle diffusion kinetic plot did not match the origin of coordinates (Figure 9c). This result indicated that the adsorption mechanism was influenced by multi-mechanisms, and that internal particle diffusion may be not the main element.

### 2.5. Thermodynamic Study

In order to understand the effect of temperature on the adsorption of MB by DFM-10 and affirm the adsorption process, thermodynamic results from the adsorption data of MB were calculated at three different temperatures by using DFM-10 (298 K, 308 K, and 318 K): Gibbs free energy Δ*G* (KJ/mol), enthalpy change Δ*H* (KJ/mol)) and entropy change Δ*S* (J/(mol·K)) [48].The calculation formula of each thermodynamic parameter is as follows:

(9) $$K_{e}=\frac{Q_{e}}{C_{e}}$$

(10) $$\Delta G=-RT\ln K_{e}$$

(11) $$\ln K_{e}=\frac{\Delta S}{R}-\frac{\Delta H}{RT}$$

where *R* is the ideal gas constant (8.314 J/mol^−1^ K^−1^). *T* acted as the absolute temperature (K). *K_e_* is the thermodynamic equilibrium constant, *Q_e_* (mg/g) is the amount of adsorption at adsorption equilibrium, and *C_e_* (mg/L) is the equilibrium concentration [49]. We plotted lnK_e_ versus 1/*T* and calculated Δ*S* from the intercept and Δ*H* from the slope. The calculation results are listed in Table 3.

It can be seen from Table 4 that Δ*H* was positive, indicating that the adsorption of MB in aqueous solution by DFM-10 was an endothermic process. The higher the temperature, the better the adsorption effect. When Δ*H* was less than 40 kJ/mol, the adsorption process was dominated by physical adsorption [50], and the Δ*H* value of the adsorption of MB in aqueous solution by DFM-10 was 4.6747 kJ/mol within this range, indicating that the adsorption process was dominated by physical adsorption. Δ*G* was negative, indicating that the adsorption process of DFM-10 to MB was spontaneous, and as the temperature increased, the value of Δ*G* gradually decreased, which shows that the spontaneity of the reaction was gradually increasing as the temperature rose. When the value of Δ*G* is in the range of −20 to 0 kJ/mol, the adsorption process is physical adsorption. Obviously, the Δ*G* value of the adsorption of MB in the aqueous solution by DFM-10 was within this range, indicating that the adsorption process was physical adsorption, which is consistent with the discussion of the Δ*H* value. In addition, Δ*S* was 16.8767 kJ/mol greater than 0, indicating that the disorder of the solid−liquid interface increased during the process of adsorption and the adsorption process was irreversible. Thermodynamic studies have shown that the adsorption of MB in aqueous solution by DFM-10 is a spontaneous endothermic physical adsorption process.

**Figure 10:**
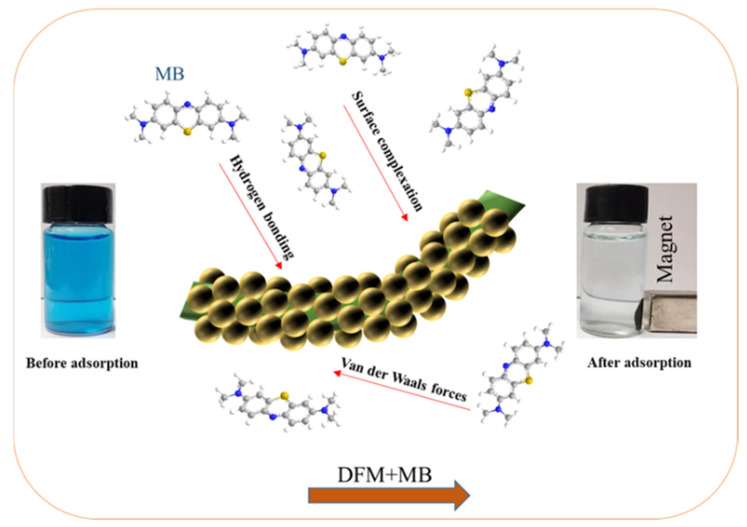
Possible adsorption mechanism of MB onto DFM-10.

Based on all the discussions above, the interaction mechanism when DFM-10 adsorbs MB is proposed, as shown in Figure 10. It can be seen from Table 4 that the specific surface area of DFM-10 is 226.88 m^2^/g, which is significantly higher than that of DFM-10. At the same time, DFM-10 has smaller mean pore radius and larger total pore volume, which provides a good structural basis for MB adsorption. Meanwhile, in the metal–organic framework (MOF) synthesis process, H_3_BTC and MB molecules have a weak interaction and can be adsorbed by hydrogen bonds and other forces [51]. The -OH structure in DFM-10 can be used as a π electron donor, and the conjugated unsaturated carbonyl structure in MB molecule can be used as a π electron acceptor. Therefore, the π−π interaction in the adsorption process may be a potential mechanism.

**Figure 11:**
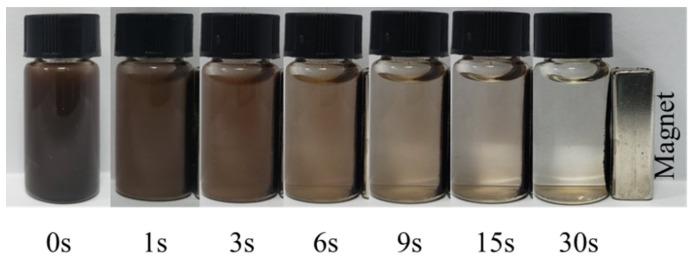
Magnetic separation process of DFM-10 in solution.

Figure 11 shows the separation performance of DFM-10 in aqueous solution. It can be seen from the same figure that under the action of a strong magnet, DFM-10 can be basically separated from aqueous solution within 9 s, and can be completely separated from aqueous solution within 30 s. This is because the load of Fe_3_O_4_ provides the ability for rapid separation of DFM-10 from aqueous solution.

### 2.6. Total Organic Carbon (TOC) Analysis

**Figure 12:**
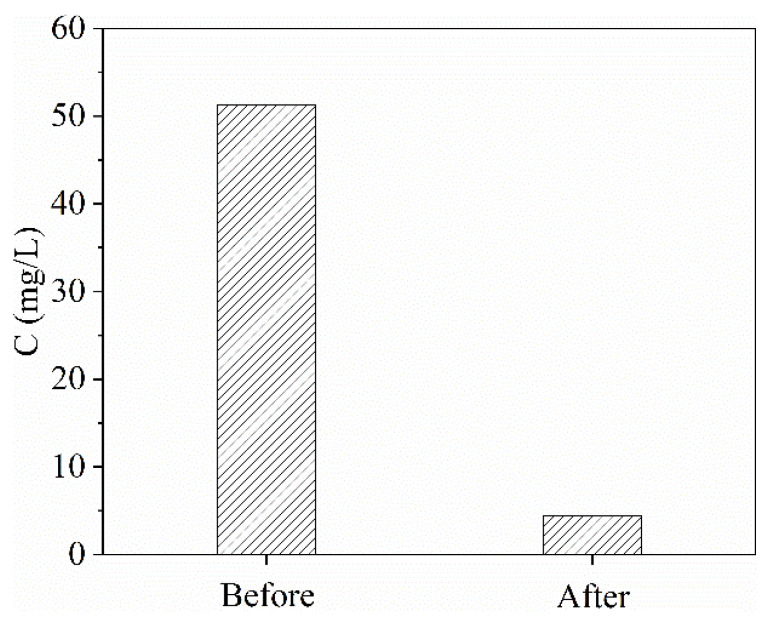
Removal effect of DFM-10 on TOC in actual wastewater.

The total organic carbon (TOC) is an indicator for evaluating the removal of organic pollutants in water. Figure 12 showed the TOC amount of the natural river water (Hung-tse Lake in Jiangsu Province, Hongze) before and after adsorption by DFM-10. The initial amount of TOC was 51.22 mg/L before adding DFM-10. After the absorption reaction, the TOC amount decreased to 4.39 mg/L. The results indicated that the TOC removal rate reached 91.44%, indicating that the adsorbent had excellent purification performance.

### 2.7. Recycling Ability

**Figure 13:**
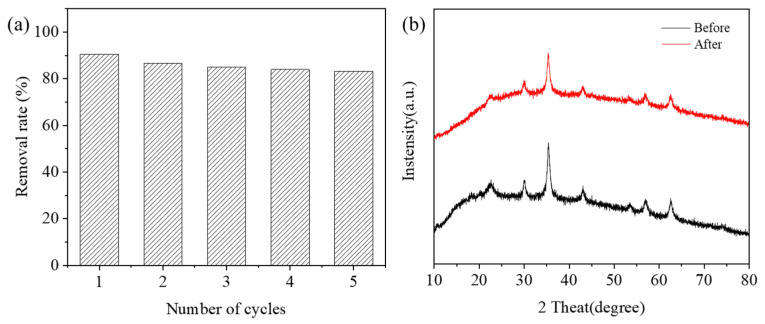
(**a**) Recycling performance of DFM-10 adsorbent and (**b**) XRD patterns of DFM-10 before and after five reaction cycles.

Through cyclic experiments, we studied whether DFM-10 can be recycled, which has an important impact on the practicality of the adsorbent. We conducted MB adsorption-cycle experiments on DFM-10 five times. At 298 K, the initial concentration of MB was 10 mg/L. After 3 h of adsorption, the adsorption was carried out with HCl/ethanol (0.5%, *v*/*v*). The obtained result is shown in Figure 13. It can be seen from Figure 13a that the removal rate of MB by DFM-10 was 90.50% in the first cycle. After five cycles, the removal rate of MB by DFM-10 remained at 83.15% without significant decrease. From Figure 13b, the crystal structure of DFM-10 had no apparent change after four consecutive cycle reactions, suggesting that DFM-10 could be used as an efficient and recyclable adsorbent to remove MB from wastewater. The removal efficiency of DFM-10 for MB was also higher than reported adsorbents, as mentioned in Table S1 [52,53,54,55,56,57].

## 3. Materials and Methods

### 3.1. Chemicals and Reagents

Durian shells were purchased from supermarket in Nanjing. Ferric chloride hexahydrate (FeCl_3_•6H_2_O) and sodium chlorite (NaClO_2_) were from Shanghai Macklin Biochemical Co., Ltd.(Shanghai, China) Benzene-1, 3, 5-tricarboxylic acid (H_3_BTC) and urea (CH_4_N_2_O) were purchased from Aladdin Reagent Co., Ltd. (Shanghai, China). Sodium hydroxide (NaOH), sodium bicarbonate (NaHCO_3_), and methylene blue (MB) were purchased from Sinopharm Chemical Regent Co., Ltd. (Shanghai, China). Vitamin C (C_6_H_8_O_6_) was purchased from Yuanye Biological Technology Co., Ltd. (Shanghai, China). All the above chemical reagents were used without further purification.

### 3.2. Synthesis of Adsorbent

#### 3.2.1. Extraction of Durian Shell Fiber

Firstly, 25 g of durian shell was dried at 60 °C for 6 h, cut into pieces, and placed in a NaOH solution (1 M, 500 mL). The solid was then stirred at 85 °C for 4 h to remove cellulose and impurities. Subsequently, the durian shell fibers were put into a 5 wt% NaClO_2_ solution for decolorization, and magnetically stirred at 80 °C for 2h, filtered, separated, washed five times with deionized water, and dried at 60 °C for 24 h to get durian shell fibers.

#### 3.2.2. Preparation of Durian Shell Fiber–Fe_3_O_4_ (DF)

Firstly, 0.2 g durian husk fiber, 2.0 g NaOH, and 4.0 g CH_4_N_2_O were dissolved in 40 mL deionized water, stirred to form a transparent solution, and flocculent fibers were collected. Secondly, 0.2 mol/L FeCl_3_•6H_2_O solution (10 mL) was added to 0.45 mol/L NaHCO_3_ solution (10 mL), and then vigorously stirred for 30 min. Then, 0.03 mol/L vitamin C solution (10 mL) was added to above-mentioned flocculent durian shell fiber and stirred for 20 min at 25 ℃. Then, the material was put into a 50 mL Teflon kettle and heated at 180 °C for 8 h. The final products could then be separated with ethanol and washed with deionized water three times, collected by centrifugation (8000 rpm) and dried at 80 °C for 12 h to obtain durian shell fiber–Fe_3_O_4_ (DF).

#### 3.2.3. Preparation of Durian Shell Fiber–Fe_3_O_4_ MOF (DFM)

**Scheme 1:**
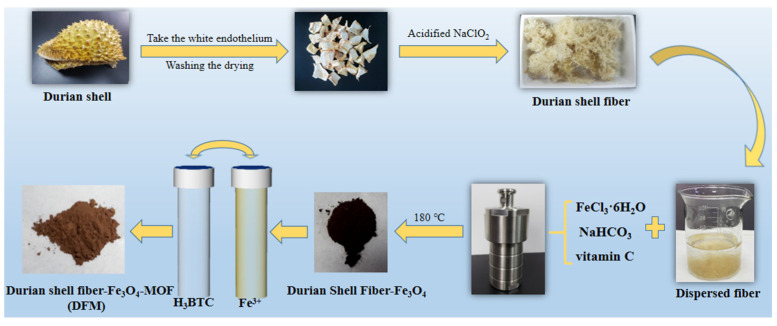
The synthetic process of DFM preparation.

The preparation of durian shell fiber–Fe_3_O_4_ MOF composite material was carried out by the self-assembly method. Firstly, 0.05g of the prepared DF was dispersed ultrasonically and added to 4 mL FeCl_3_•6H_2_O ethanol solution (10 mmol/L) for 15 min, then dispersed in 4 mL of H_3_BTC ethanol solution (10 mmol/L), and heated at 70 °C for 30 min. Each step was separated with a strong permanent magnet and washed with ethanol. After a certain number of FeCl_3_•6H_2_O/H_3_BTC cycles, the sample was recovered with a permanent magnet and washed with ethanol/water, and then vacuum dried at 60 °C for 12 h to obtain DFM. The synthetic process of DFM preparation can be seen in Scheme 1. The obtained samples at different cycles (0, 10, 20, and 40) were named DF, DFM-10, DFM-20, and DFM-40, respectively.

### 3.3. Adsorption Properties

In the adsorption experiment, the MB stock solution had a concentration of 100 mg/L. It was stored in a dark place and dilute to 2.5–20 mg/L when used. The MB adsorption experiment was carried out in a constant-temperature shaker. The specific experiment was as follows: The adsorbent (5 mg) was mixed with 15mL MB solutions of different initial concentrations and placed in constant-temperature oscillator at 25 °C. After a reaction time of 3 h, the adsorbent material was collected with a magnet. MB concentration in the supernatants was measured at 664 nm by using a UV–Vis spectrophotometer. The quantity of adsorption (Q_e_ (mg/g)) and rate of removing MB (R (%)) were calculated based on the following equations:

(12) $$Q_{e}=\frac{\left(C_{0}-C_{e}\right)\times V}{m}$$

(13) $$R=\frac{C_{0}-C_{e}}{C_{0}}\times 100\%$$

where *C_0_* (mg/L) is the initial concentrations of MB and *C_e_* (mg/L) is the equilibrium concentrations of MB; *m* is determined to the weight of adsorbent (g), and *V* is defined as the volume of the liquid solution (mL) [58,59].

### 3.4. Characterization

FTIR spectra of the sample was performed with an FTIR spectrometer (AVATAR 360, Nicole company, MA, USA). A minimum of 32 scans was signal-averaged with a resolution of 2 cm^−1^ in the 4000–400 cm^−1^ ranges. X-ray diffraction (XRD) analysis of the material was conducted on X-ray powder diffraction (XRD-6100, Shimadzu company, Kyoto, Japan) with a scanning rate of 5 °/min. The morphology of the as-prepared adsorbent was characterized by scanning electron microscope (SEM, S-4800, Hitachi company, Kyoto, Japan). Transmission electron microscopy (TEM) analysis was undertaken on a JEM-1400 UV–Vis spectrophotometer (UV-2450, Shimadzu Company, Kyoto, Japan). The zeta potential of material was tested by using a Zeta potential analyzer (Zetasizer Nano ZS90, Malvern Instruments Ltd., Malvern, UK). Total organic carbon(TOC) was obtained by total organic carbon analyzer (Shimadzu, Kyoto, Japan).

## 4. Conclusions

In this work, durian shell biomass carbon fiber and Fe_3_O_4_ functionalized metalorganic framework composite material (DFM) was prepared and the structure was characterized. Compared with conventional adsorbents, it can effectively remove MB from wastewater. Then, comparing the adsorption capacity with different adsorbents to MB, it can be seen that DFM-10 had the best effect and the maximum adsorption capacity for MB was 53.31mg/g. Adsorption isotherms studies showed that DFM-10 is more in line with the Langmuir isotherm model, adsorption kinetics studies showed that DFM-10 is more in line with the pseudo-second-order kinetic model, and thermodynamic analysis showed that DFM-10 adsorbs MB in an aqueous spontaneous endothermic physical adsorption process. This study proposes an effective method to prepare DFM composite material, which has high adsorption efficiency and can be recycled, and is expected to be widely used to removing MB in wastewater.

## Figures and Tables

**Table 1:** Langmuir, Freundic, Temkin, and Dubinin–Radushkevich adsorption isotherm model parameters.

Model	Parameter	298 K	308 K	318 K
Langmuir	*Q_l_* (mg/g)	34.8100	42.2500	53.3100
	*K_L_* (L/mg)	1.8427	1.3402	1.9642
	*R* ^2^	0.9931	0.9948	0.9791
Freundich	*K_F_* (mg^1−1/n^ L^1/n^ /g)	20.5600	22.6900	30.6800
	1/*n*	0.2065	0.2624	0.2862
	*R* ^2^	0.9766	0.9609	0.9570
Temkin	*b_T_* (J.g/mol.mg)	0.7053	0.5124	0.4142
	*K_T_* (L/mg)	1.0065	1.0051	1.0054
	*R* ^2^	0.8910	0.8735	0.8447
Dubinin–Radushkevich	*Q_l_* (mg/g)	28.3731	31.8753	40.1395
	*K_DR_* (molJ)	0.1105	0.1432	0.1423
	*R^2^*	0.8802	0.8567	0.8811

**Table 2:** Pseudo-first-order, pseudo-second-order, and intraparticle diffusion dynamics-model-related parameters.

Model	Parameter	5 (mg/L)	10 (mg/L)	15 (mg/L)
Pseudo-first-order	*Q_e_* (mg/g)	1.5100	4.0900	6.8100
	*K*_1_ (/min)	0.0225	0.0301	0.0438
	*R* ^2^	0.6817	0.8041	0.9523
Pseudo-second-order	*Q_e_* (mg/g)	15.0900	27.6000	37.5100
	*K_2_* (g/(mg min))	0.0581	0.0171	0.0101
	*R* ^2^	0.9998	0.9998	0.9998
	*K_p_*_1_ (mg/g/min^1/2^)	0.3237	0.3767	0.7017
	*C*_1_ (mg/g)	3.5209	6.7550	9.4854
	*R* ^2^	0. 9961	0. 9928	0.9884
Intraparticle diffusion	*K_p_*_2_ (mg/g/min^1/2^)	0.0184	0.0993	0.1144
	*C*_2_ (mg/g)	4.7022	8.3343	12.6905
	*R* ^2^	0. 8202	0.7468	0.5608
	*K_p_*_3_ (mg/g/min^1/2^)	0.0230	0.1345	0.0730
	*C*_3_ (mg/g)	4.6616	7.9615	13.2741
	*R* ^2^	0. 9665	0.9591	0. 9548

**Table 3:** Thermodynamic parameters for MB adsorption by DFM-10.

Δ*H*	Δ*S*	Δ*G* (kJ/mol)			*R* ^2^
(KJ/mol)	(J/(mol·K))	298 K	308 K	318 K	
4.6747	16.8786	−3.0115	−4.2291	−5.8263	0.9851

**Table 4:** Porous performance parameters of DF and DFM-10.

Simples	DF	DFM-10
Mean pore radius (nm)	10.53	5.45
Total pore volume (cm^3^ g^−1^)	0.19	0.24
BET surface area (m^2^ g^−1^)	60.55	226.88

## Data Availability

The data presented in this study are available on request from the corresponding author.

## Supplementary Materials

The following supporting information can be downloaded at https://www.mdpi.com/article/10.3390/ijms23115900/s1. References [52,53,54,55,56,57] are cited in supplementary materials.

## Sample Availability

Samples of the compounds are not available from the authors.
