# Supplementary material for: Waste-to-Resource Strategy to Fabricate Functionalized MOFs Composite Material Based on Durian Shell Biomass Carbon Fiber and Fe3O4 for Highly Efficient and Recyclable Dye Adsorption
Source: Int J Mol Sci. 2022 May 24;23(11):5900. doi: 10.3390/ijms23115900 (PMC9180916; doi:10.3390/ijms23115900)
Supplement: Supplementary file 1 [file ijms-23-05900-s001.zip › ijms-1674588-supplementary.pdf]

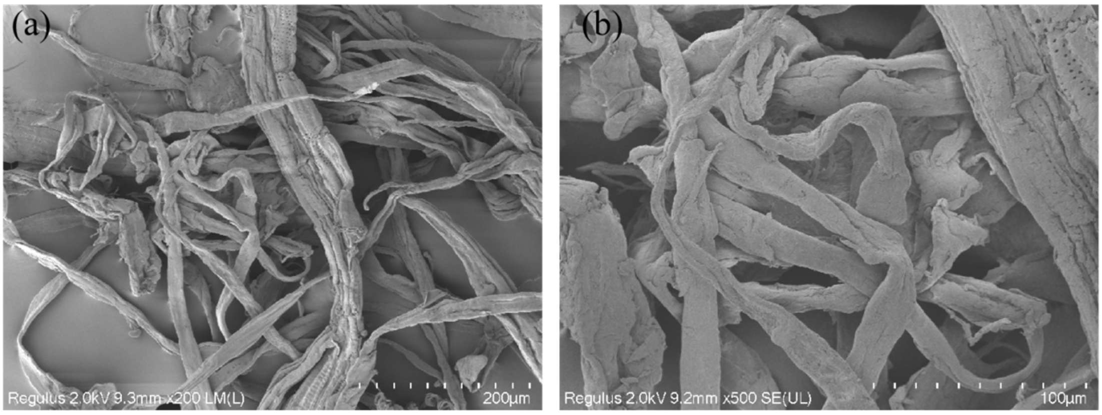

Figure S1. SEM images of durian shell fiber

Table S1. Previous studies on the adsorption of MB with different adsorbents.

Adsorbents	Temperature(K)	Q_e（mg/g）	Ref.
Chitosan-crosslinked bismuth ferrite/biochar	298	18.942	[52]
BC-HAP	313	21.1	[53]
Acrylic acid–acrylonitrile–N-isopropylacrylamide polymeric gels	298	2.79	[54]
Biochar	298	7.2	[55]
PG	298	0.2	[56]
Eichhornia crassipes biomass	298	35.4	[57]
DFM-10	298-318	53.31	This work

Reference:

52. Cai, X.; Li, J.; Liu, Y.; Hu, X.; Tan, X.; Liu, S.; Wang, H.; Gu, Y.; Luo, L. Design and Preparation of Chitosan-Crosslinked Bismuth Ferrite/Biochar Coupled Magnetic Material for Methylene Blue Removal. *Int. J. Environ. Res. Public Health.* **2020**, *17*, 6. https://doi.org/10.3390/ijerph17010006.
53. Li, Y., Zhang, Y., Zhang, Y., Wang, G., Li, S., Han, R., Wei, W., Reed biochar supported hydroxyapatite nanocomposite: Characterization and reactivity for methylene blue removal from aqueous media. .*J. Mol. Liq*. **2018**, *263*, 53–63. https://doi.org/10.1016/j.molliq.2018.04.132.
54. Aslam Malana, M., Parveen, S., Beenish Qureshi, R., Adsorptive removal of organic dyes from aqueous solutions using acrylic acid-acrylonitrile-N-isopropylacrylamide polymeric gels as adsorbents: linear and non linear isotherms. *DESALINATION Water Treat*. **2016**, *57*, 22543–22550. https://doi.org/10.1080/19443994.2015.1132393.
55. Jh, A., Hg, B., Nm, A., Hj, A., Removal of methylene blue from aqueous solutions by biochar prepared from the pyrolysis of mixed municipal discarded material. *Sci. Total Environ*. **2020**, *714*, 136832. https://doi.org/10.1016/j.scitotenv.2020.136832.
56. Gao, Y., Sun, D., Han, C., Huang, J., Comprehensive Utilization of Phosphogypsum: Adsorption of Methylene Blue and its Application in Bricks. Surf. Rev. Lett. **2021**, *28*, 2150075. https://doi.org/10.1142/S0218625X2150075X.
57. Wanyonyi, W. C. , Onyari, J. M. , Shiundu, P. M., Adsorption of methylene blue dye from aqueous solutions using eichhornia crassipes. Bulletin of Environmental Contamination & Toxicology. **2013**, *91*, 362-366. https://doi.org/10.1007/s00128-013-1053-0.
